# Assessment of Heparanase-Mediated Angiogenesis Using Microvascular Endothelial Cells: Identification of λ-Carrageenan Derivative as a Potent Anti Angiogenic Agent

**DOI:** 10.3390/md15050134

**Published:** 2017-05-09

**Authors:** Nicolas Poupard, Pamela Badarou, Fabienne Fasani, Hugo Groult, Nicolas Bridiau, Frédéric Sannier, Stéphanie Bordenave-Juchereau, Claudine Kieda, Jean-Marie Piot, Catherine Grillon, Ingrid Fruitier-Arnaudin, Thierry Maugard

**Affiliations:** 1 Université de la Rochelle, UMR CNRS 7266, LIENSs, Equipe Approches Moléculaires, Environnement-Santé, Avenue Michel Crépeau, 17000 La Rochelle, France; nicolas.poupard@univ-lr.fr (N.P.); hugo.groult@univ-lr.fr (H.G.); nicolas.bridiau@univ-lr.fr (N.B.); frederic.sannier@univ-lr.fr (F.S.); stephanie.bordenave@univ-lr.fr (S.B.-J.); jean-marie.piot@univ-lr.fr (J.-M.P.); thierry.maugard@univ-lr.fr (T.M.); 2 Centre de Biophysique Moléculaire, UPR CNRS 4301, 45071 Orléans, France; ayobadarou@hotmail.fr (P.B.); fabienne.fasani@cnrs-orleans.fr (F.F.); claudine.kieda@cnrs-orleans.fr (C.K.); catherine.grillon@cnrs-orleans.fr (C.G.)

**Keywords:** heparanase, angiogenesis, endothelial cells, λ-Carrageenan, hypoxia, anti-angiogenic, sulfated polysaccharide

## Abstract

Heparanase is overexpressed by tumor cells and degrades the extracellular matrix proteoglycans through cleavage of heparan sulfates (HS), allowing pro-angiogenic factor release and thus playing a key role in tumor angiogenesis and metastasis. Here we propose new HS analogs as potent heparanase inhibitors: Heparin as a positive control, Dextran Sulfate, λ-Carrageenan, and modified forms of them obtained by depolymerization associated to glycol splitting (RD-GS). After heparanase activity assessment, 11 kDa RD-GS-λ-Carrageenan emerged as the most effective heparanase inhibitor with an IC_50_ of 7.32 ng/mL compared to 10.7 ng/mL for the 16 kDa unfractionated heparin. The fractionated polysaccharides were then tested in a heparanase-rich medium-based in vitro model, mimicking tumor microenvironment, to determine their effect on microvascular endothelial cells (HSkMEC) angiogenesis. As a preliminary study, we identified that under hypoxic and nutrient poor conditions, MCF-7 cancer cells released much more mature heparanase in their supernatant than in normal conditions. Then a Matrigel^TM^ assay using HSkMEC cultured under hypoxic conditions in the presence (or not) of this heparanase-rich supernatant was realized. Adding heparanase-rich media strongly enhanced angiogenic network formation with a production of twice more pseudo-vessels than with the control. When sulfated polysaccharides were tested in this angiogenesis assay, RD-GS-λ-Carrageenan was identified as a promising anti-angiogenic agent.

## 1. Introduction

It is now well known that solid tumor growth beyond 1–2 mm^3^ is dependent on angiogenesis, the sprouting of new blood vessels from preexisting ones to perfuse the tumor [1]. This process promotes the tumor access to the nutrients and oxygen required for its development, opening in the meantime, the way to metastasis. Tumor angiogenesis is triggered by a range of pro-angiogenic factors released in the tumor microenvironment such as enzymes, chemokines and growth factors (VEGF and FGFs) that interact with surrounding blood vessels and induce neovascularization. It has been shown that hypoxia, the condition where cells suffer from oxygen deprivation, particularly stimulates the expression of these factors [2,3].

Considering the poor prognosis associated with tumor neovascularization, anti-angiogenic therapies have become an attractive approach to cancer therapy [4]. Anti-angiogenic treatments leading to excessive blood vessels pruning or disruption seem to induce the selection of aggressive and resistant cancer phenotypes because they intensify hypoxia and activate the production of even more pro-angiogenic factors [5]. Nowadays, clinicians prefer to normalize angiogenesis to create a proper matured blood flow that reduces hypoxia, the occurrence of edema caused by leaky vessels and cell spreading while enhancing drug delivery efficiency [6].

In particular, growth factors determine the development of angiogenesis and thus have become a target of choice for its normalization [7]. Treatments using inhibitors such as anti-VEGF antibodies (Avastin^®^) are useful tools, when correctly administrated, for VEGF modulation rather than total inactivation. Approaches using VEGF trap have also shown their efficiency for angiogenesis normalization and tumor growth inhibition [8]. However, these approaches may be faced with other problems, including “tumor evasion”, where the tumor adapts and produces other derived pro-angiogenic factors to compensate the VEGF neutralization [9]. Thus, targeting degradative enzymes present in the tumor microenvironment and associated with angiogenesis should be an effective strategy to develop new anti-angiogenic treatments.

Among them, heparanase, an endo-β-d-glucuronidase, plays a crucial role in tumor development, metastasis and angiogenesis [10,11]. It is involved in extracellular matrix (ECM) and vascular basement membrane degradation, allowing tumor cells to spread and/or endothelial cells to form new blood vessels in order to perfuse the tumor. In the ECM, heparan sulfates (HS) are cleaved by heparanase which induces, as a consequence, the release of numerous pro-angiogenic chemokines and growth factors, including VEGF and FGF-2, in the tumor microenvironment [12]. Since the gene encoding heparanase has been cloned [13], the enzyme has been more extensively studied and heparanase is now considered as a potential target for new anti-angiogenic treatments [14].

To date, several heparanase inhibitors from different chemical classes have been developed [14]. Among them, heparan sulfate mimics, such as PI88 [15], PG545 [16], and SST0001 [17], emerged as good candidates and are currently in different phases of clinical trials. The latter, SST0001 (roneparstat, Sigma-Tau research, Switzerland SA, Mendrisio, Switzerland) is a non-anticoagulant 100% N-acetylated heparin with a 25% “glycol split” [18]. Glycol-splitting, a two-step reaction involving an oxidation then a reduction, is of particular interest for heparins as it opens hexuronic rings and disrupts their anticoagulant activities. More interestingly, this process seems to give more flexibility to the polysaccharide chains and enhances the inhibition of heparanase [19]. Recent reports emphasized the difficulty to find safe and reliable sources of heparin [20], underlining the need to study alternative supply sources of sulfated polysaccharides. Interestingly, certain plants or seaweeds can produce polysaccharides structurally close to HS [21,22]. Dextran sulfate (shortened DextranS in the paper) are highly sulfated polysaccharides that can easily be produced by hypersulfation of dextran produced by bacteria (e.g., *leuconostoc mesenteroides*). λ-Carrageenan can be extracted from red marine algae species. They are widely used in the food-industry as thickening agent [23] and already showed interesting results as anti-angiogenics [24]. Raw unfractionated (UF) products from λ-Carrageenan or dextranS can have high molecular weights up to millions of Daltons. To limit side effects and increase bioavailability of these compounds, a depolymerization process is necessary to obtain species with low molecular weights (LMW), less than 15 kDa. We previously described a radical-assisted ultrasonic (RD) method to depolymerize sulfated polysaccharides [25] and decided to associate it with glycol splitting (GS) to give higher flexibility to the chains.

The main objective of this study was then to assess the anti-heparanase activities of these products along with their anti-angiogenic potential.

One of the major outcomes when identifying anti-angiogenic drugs is the relevance of in vitro angiogenesis models used for bioassays. The model shall be easy, repeatable, close to the in vivo conditions with accessible information about the metabolic pathways that the drug will affect. To date, plethora of models have been proposed and one of the most popular consists in cultivating endothelial cells in a 3D Matrigel^TM^ composed of mouse tumor-derived ECM. Using this test, endothelial cells are able to develop capillary-like structures with cell extensions resembling chords [26]. It is particularly adapted for the study of ECM degradation and evaluation of anti-angiogenic agents. However, this test has often been used with macrovascular endothelial cells (HUVECs for instance) that may not be the most representative model of tumor neovascularization, mostly composed of microvasculature [27].

Thus, a second aspect of our work was to develop a Matrigel^TM^ based model to specifically investigate the role of heparanase on microvascular endothelial cell angiogenesis in hypoxic conditions. The HSkMEC cell line was chosen because of its microvascular profile with a stable phenotype in terms of adhesion molecules [28] and typical endothelial cell characteristics (presence of angiotensin-converting-enzyme and von Willebrand factor [29]). The exposition of HSkMEC cells cultured in Matrigel^TM^ to tumor microenvironment conditions such as hypoxia or nutrient deprivation was studied regarding the angiogenesis behavior and heparanase production. The role of an exogenous source of heparanase was also evaluated via the addition of heparanase-rich supernatants in media of HSkMEC cell cultures. The latter was produced from hypoxic cultures of breast carcinoma cells.

Using this in vitro angiogenesis assay that comprises heparanase and tumor microenvironment conditions, it was possible to screen potential anti-angiogenic candidates among the depolymerized and glycol split (RD-GS) HS mimicking compounds that we generated from UF-heparins, UF-dextranS and UF-λ-Carrageenan.

## 2. Results and Discussion

### 2.1. Preparation and Characterization of Sulfated Polysaccharides

To produce a range of modified sulfated polysaccharides, we used UF-λ-Carrageenans, UF-heparins and UF-dextranS as starting material. 

Heparins are certainly the most studied HS mimics and their role in the regulation of the angiogenic process is well known [30]. Moreover, the inhibition of heparanase by heparins and their derivatives has already been explored. It helped to further understand the mechanism of action of the endo-glycosidase [19,31,32] revealing the importance of sulfate groups and the sulfation pattern responsible for its inhibition. Although heparins are interesting heparanase inhibitors, they are currently extracted from porc intestines in low yields. This, added to the drastic and costly purification controls, emphasize the needs for alternative inhibitors [33].

**Figure 1:**
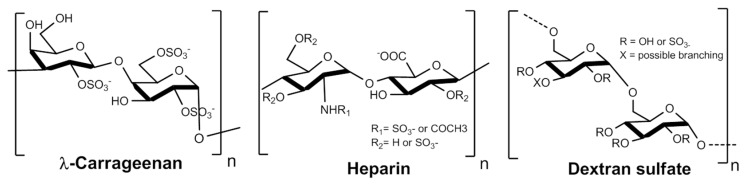
Structure of the three sulfated polysaccharides studied: λ-Carrageenan, Heparin and Dextran sulfate. Concerning the heparin structure, the glucuronic acid may be epimerized as iduronic acid along the chain and bear an O-sulfatation on the C-3 position.

λ-carrageenans are sulfated polysaccharides naturally occurring in seaweed species such as *Gigartina* [34] and dextranS can be easily produced by hypersulfation of dextran extracted from bacteria (e.g., *leuconostoc mesenteroides*). We selected these polysaccharides because their structures (Figure 1) have high similarities with HS and because they could provide a reliable source of sulfated polysaccharides from biomass. Still, heparins will be used in this study as a reference.

One major problem with polysaccharide extracts is the size of the chains, which are often long and generate side effects when used as therapeutics. To resolve this, a depolymerization step is necessary. Depolymerization reduces chain length, enhances polysaccharide bioavailability and helps to modulate some biological activities [35].

The three polysaccharides studied here were submitted to depolymerization (RD) using a previously described method which associates radicals (from hydrogen peroxide) and ultrasonic waves [36]. After depolymerization, the compounds were submitted to glycol splitting (GS) [18] (Sodium periodate oxidation followed by sodium borohydride reduction) and then respectively called RD-GS-Heparin, RD-GS-DextranS and RD-GS-λ-Carrageenan.

Glycol splitting is particularly interesting in the case of heparin as it reduces its anticoagulant activity [37]. It also increases chain flexibility with the opening of C2–C3 unsubstituted uronic acid rings which is thought to increase heparanase inhibition [19]. We thus proposed it would be of interest to combine depolymerization and glycol splitting to modulate the biological activities of our three sulfated polysaccharides. The unfractionated structures of dextranS and λ-carrageenan do not possess vicinal diols susceptible to be oxidized by the glycol-split process. However, radical depolymerization assisted by ultrasounds can induce a loss of sulfate functions [25,38] in addition to the shortening of the polysaccharides chain length. We then hypothesized it may produce derivatives with hexoses possessing C2–C3 diols, rendering them sensitive to glycol-splitting. The opening of the hexose rings would thus offer a subsequent gain in chain plasticity.

The extent of sulfation of polysaccharides was analyzed by Azure A colorimetric assay on products solutions and compared to a calibration curve using dextran sulfate with 17% sulfur (*w*/*w*). Comparing UF-Heparin and RD-GS Heparin, a clear decrease of 0.6 in the sulfation degree was observed after depolymerization and glycol splitting (see Table 1). RD-GS-λ-Carrageenans have a sulfation degree of 1.1 close to the lowest degree of 0.9 observed for RD-GS-DextranS. The number average molecular weight (M*_n_*) of each compound was measured using size exclusion chromatography. The size of RD-GS products was inferior to 5000 Da for heparins and dextranS when the size of RD-GS-λ-Carrageenans were around 11,000 Da.

Using an enzyme assay kit, the degradation of biotinylated heparin by heparanase in the presence of potent inhibitors was studied (Table 1). As expected, a low concentration (10.7 ng/mL) of UF-Heparin was needed to inhibit 50% of the heparanase activity. Comparatively, RD-GS-λ-Carrageenan appeared as a better heparanase inhibitor with an IC_50_ of 7.32 ng/mL while the IC_50_ of RD-GS-DextranS was moderate at 61.5 ng/mL. As observed, comparing UF-Heparin IC_50_ (10.7 ng/mL) and RD-GS-Heparin IC_50_ (147 ng/mL), the combination of depolymerization and glycol splitting decreased the anti-heparanase activity of the unfractionated polysaccharide. The same observations were made in a recent study comparing UF-species of λ-Carrageenan and their RD-GS counterparts [38]. A new study on heparanase crystal structure revealed that the enzyme binding cleft is lined by basic side chains, explaining the huge importance of sulfation pattern for substrate recognition [39]. Moreover, in the case of heparin, it has been shown that around the cleavage site, the 2-N sulfation and 6-O sulfation of motifs in −1 and +1 positions are mandatory for recognition. The RD-GS process may have altered the heparin 2-N or 6-O sulfation pattern more than the λ-carrageenan D2S,6S-G2S one, and could explain the better inhibition observed for RD-GS-λ-Carrageenans. This suggests that beside a useful depolymerization effect, the RD-GS process did not impair the structure of λ-Carrageenans to a point where it is not recognized by heparanase. RD-GS-DextranS have a low sulfation degree of 0.9 that could account for their weaker inhibition of heparanase and RD-GS-Heparin with a sulfation degree of 1,4 displayed an even weaker inhibition of the enzyme. It has been hypothesized that a reasonably long polysaccharide can bind two enzymes at the same time and create inhibition [40]. RD-GS-DextranS, RD-GS-λ-Carrageenan and UF-Heparin have longer chains (4710 Da, 11,396 Da and 16,412 Da, respectively) than RD-GS-Heparin (2716 Da) and may bind two enzymes at the same time. These results support the fact that sulfation pattern of a polysaccharide alone cannot create an inhibition without a proper chain length.

The HSkMEC viability was then tested by exposing cells for 48 h to the compounds at the concentration of 250 µg/mL in a complete OPTIMEM medium with 2% added FBS. Despite the toxicity of UF-heparins that decreased cell viability to 68.9 ± 13.9%, the RD-GS derivatives showed relatively low impact on cell viability, diminishing it by less than 16%. These data indicated that the effects of these compounds on the formation of the angiogenic network (shown later in Figure 4) could not be misinterpreted with a potent cytotoxicity.

The presented cell viability data were part of a dose dependent study realized for UF-Heparins, RD-GS-Heparin and RD-GS-λ-Carrageenan from 0 to 500 µg/mL [38]. This study showed a correlation between higher compounds concentrations and higher cytotoxicities, UF-heparin being the most hazardous for HskMEC cells and RD-GS-λ-Carrageenan being the least.

### 2.2. Expression and Secretion of Heparanase in Human Skin Microvascular Endothelial Cells and Human Mammary Cancer Cells

In order to assess the possible heparanase-related angiogenesis induced by tumors, we first studied heparanase expression and secretion in the endothelial cell line HSkMEC and in different breast cancer cell lines. Heparanase is known to be overexpressed in the breast carcinoma microenvironment [41], so breast cancer cell lines producing heparanase-rich supernatant were selected. The heparanase expression profiles of non-metastatic MCF-7 breast carcinoma cells and moderate metastatic MDA-MB-231 breast carcinoma cells were assessed and compared. Because we wanted to study the effects of tumor microenvironment drastic conditions on heparanase expression, cells were cultured in hypoxia and in nutrient poor conditions (FBS deprived).

**Figure 2:**
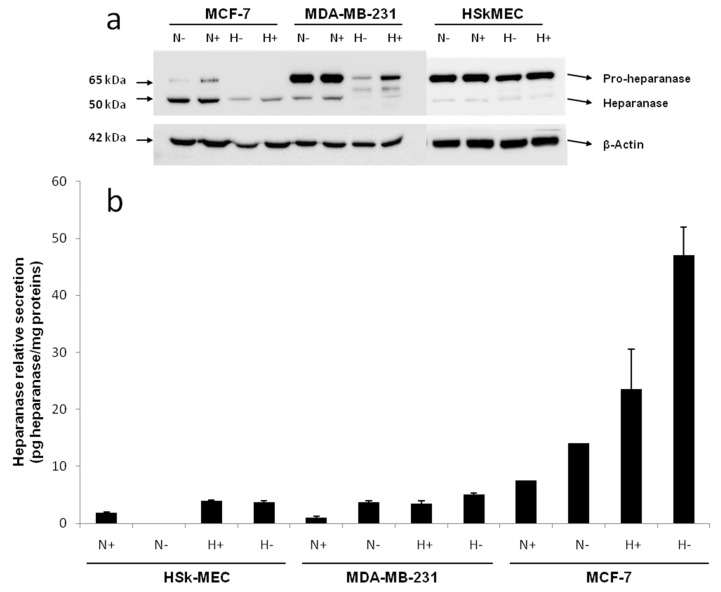
Determination of heparanase expression in both cell lysates and medium of HSkMEC, MDA-MB-231 and MCF-7 cells. (**a**) Western blot analysis of cell lysates (22.5 µg of total proteins) obtained from cells cultured under hypoxic or normoxic conditions for 48 h in 2% FBS or FBS-free medium. Bands were reported relative to the level of β-Actin. (**b**) ELISA quantification of heparanase in cell supernatant obtained from cells cultured under hypoxic or normoxic conditions for 48 h in 2% FBS or FBS-free medium. H indicates Hypoxia; N indicates Normoxia; “+” indicates that 2% FBS was present in the medium; “−“ indicates that the medium was FBS-free. Results are expressed as a relative heparanase release compared to the protein content in the cell lysate. Results are presented as the mean ± SD for triplicates.

By Western blotting, the endogenous level of heparanase in the cell lysate of the three studied cell lines was determined after a 48 h incubation in 2% FBS or FBS-free medium under normoxic (95% air, 5% CO_2_) and hypoxic (1% O_2_) conditions (Figure 2a).

Western blotting revealed the presence of a 65 kDa form of heparanase corresponding to the pro-enzyme and a 50 kDa form corresponding to its mature active form. In fact, heparanase is synthesized as a 543 aa/65 kDa pro-form that is activated after proteolytic cleavage. The enzyme has been reported to be a heterodimer consisting of the 50 kDa subunit (Lys^158^-Ile^543^) non-covalently associated with a 8 kDa peptide (Gln^36^-Glu^109^), with an intervening 6 kDa peptide (Ser^110^-Gln^157^) being excised by proteolysis [42]. It has been shown that the proteolytic cleavage is performed by the endopeptidase Cathepsin L in the lysosomal compartment [43].

In HSkMEC lysates, the heparanase pro-form content was high, both under hypoxia and normoxia and the mature form seemed slightly expressed. The presence of FBS did not impact the enzyme production. Concerning MCF-7 lysate, the production of mature heparanase is clearly favored in normoxia. Still, small amounts of pro-heparanase were produced in normoxia and the addition of FBS tended to increase this production. In the same way, hypoxia only showed the production of the mature form of heparanase with an increased amount when FBS is added. Beyond the hypothesis that, in hypoxia, all the pro-form was processed into the mature form, it could be assumed that for MCF-7, the pro-heparanase was also redirected to the cell surface. In fact, it has been shown that heparanase does not need to be active to play a role in cancer progression [44].

MDA-MB-231 lysates contained both forms of heparanase. The pro-form appeared to be the most expressed form both under hypoxia and normoxia and the presence of FBS in both conditions stimulated the production of the two forms of the enzyme.

The endogenous heparanase content in cell lysates (Western blot) was then compared to the content released in culture supernatant (ELISA, 50 kDa mature form specific) (Figure 2b). The first thing to note is that MCF-7 cells excreted heparanase’s mature form in its supernatant. For MCF-7 cells, the most favorable conditions for heparanase excretion appeared to be in hypoxia without added FBS as they showed a secretion 10 times higher compared to the other cell lines. The fact that less mature heparanase was detected in the lysate in hypoxia could be explained by the observed excretion of the enzyme in the same condition of culture.

Concerning HSkMEC and MDA-MB-231 cells, even if the quantity detected was low, the mature heparanase was also excreted by these cells. As for MCF-7 cells, the preferred conditions to excrete the enzyme are in hypoxia and the absence of FBS also seemed to have an impact. These results confirm not only the already known potential of endothelial cells to produce heparanase [45] but also that invasive breast cancer phenotypes such as MDA-MB-231 favor the excretion of heparanase [46]. Moreover, heparanase protein is released in the supernatant when conditions are harsh, stressful and when low oxygen concentrations are present supporting the fact that heparanase is expressed when angiogenesis is needed.

Regarding MDA-MB-231, hypoxia lowered the mature enzyme level in the lysate but this was not compensated by a high release of the enzyme in the supernatant, as seen for MCF-7. This could be explained by the fact that the mature enzyme remained attached on the cell surface even after lysis. It has been shown that heparanase may be released at the cell surface for degrading neighboring ECM or cell basement membrane [10]. Furthermore, heparanase may remain on the cell surface to activate some receptors, modifying the cell behavior through the production of more degradative enzymes [44].

Overall, these results showed that hypoxia was likely to trigger heparanase trafficking, either for its release in the supernatant or potentially to direct it to the cell surface. FBS deprivation also seemed to trigger the excretion of heparanase. This first study revealed that hypoxic conditions in FBS-free medium are the best culture conditions for MCF-7 cells to excrete high amounts of mature heparanase.

### 2.3. Effect of MCF-7 Heparanase-Rich Supernatant on HSkMEC Pseudovessels Formation

After determining the heparanase expression profile of the three studied cell lines, we developed an in vitro model to better understand the role of heparanase in tumor angiogenesis. For this purpose, we used the HSkMEC microvascular endothelial cell line capacity to form a capillary network in Matrigel^TM^. This kind of in vitro angiogenesis tests is often realized in normoxic conditions (75% N_2_, 20% O_2_, 5% CO_2_), whereas intratumoral levels of oxygen are reported to be way below the normoxic level, corresponding to the state of hypoxia [47]. Realizing experiments in normoxic conditions may induce an oxidative stress in cells that is not representative of the tumor microenvironment. To address this issue, angiogenesis tests were realized in hypoxic conditions.

We studied the effect of the addition of MCF-7 heparanase-rich supernatant produced under hypoxic conditions in FBS-free medium, i.e., under the optimal conditions previously determined to produce the highest heparanase content.

Indeed, adding MCF-7 heparanase-rich supernatant could help us investigate how heparanase affects angiogenesis in association to the heterogeneous mix of cytokines/chemokines also produced by MCF-7 breast carcinoma cells [48].

Photographs of HSkMEC hypoxic Matrigel^TM^ cultures in FBS-free medium alone and in the presence of MCF-7 heparanase-rich supernatant were taken after 9.5 h of incubation (Figure 2). The angiogenesis network was much more developed and structured in the presence of MCF-7 heparanase-rich supernatant (Figure 2b) than with FBS-free medium alone (Figure 2a). These high stress conditions triggered the release of mature heparanase whose degradative activity on Matrigel^TM^ could account for the more developed vascularization network seen in Figure 2b. Though, other chemokines and growth factors expressed in MCF-7 heparanase-rich supernatant under stress conditions may also play a role in the denser vascularization [49]. These results support the work of Cohen et al. that describe the pro-angiogenic activity of normoxic MCF-7 cells transfected with heparanase cDNA [50].

**Figure 3:**
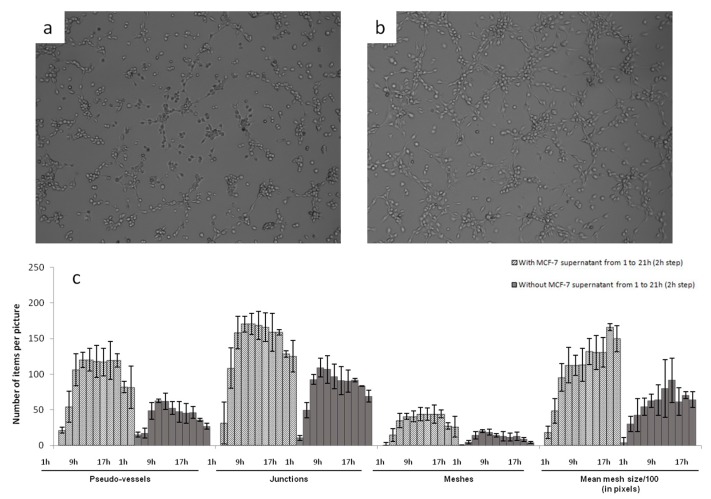
Effect of the addition of MCF-7 heparanase-rich supernatant on HSkMEC pseudo-vessels formation. Cells were plated in Matrigel^TM^ in the presence (or absence) of 10 µL of heparanase-rich supernatant from MCF-7 cultured in FBS-free medium under hypoxia for 48 h. Capillary network formation was visualized using a Zeiss Axiovert 200 M fluorescent inverted microscope and monitored for 21 h. Angiogenesis was observed after 9.5 h under hypoxia in FBS-free medium: in the absence (**a**); or in the presence (**b**) of heparanase-rich MCF-7 supernatant. (**c**) Angiogenesis formation was followed over time with pictures taken every 2 h and by studying the number of pseudovessels, junctions, meshes and the size of the meshes (indicated in pixels). Results are presented as the mean ± SD for triplicates.

After these first observations, we evaluated the behavior of vascularization development. The kinetic of angiogenesis formation was studied over 21 h by time laps microscopy. Images taken every 2 h were analyzed and treated with the ImageJ software to determine the number of pseudo-vessels, inter-vessel junctions, meshes and mean mesh size. Results are presented in Figure 3c.

This kinetic study clearly showed that adding MCF-7 heparanase-rich supernatant increased the capillary network formation. The number of pseudo-vessels formed was approximately two times higher with MCF-7 heparanase-rich supernatant than with FBS-free medium alone. The number of junctions and that of meshes were multiplied by 1.5 and 2, respectively and the mean mesh size was multiplied by 1.5. We also observed that, regardless of the presence of heparanase-rich supernatant, the number of pseudo-vessels, inter-vessel junctions and meshes formed, first tended to increase between 0 to 7 h of incubation, then reached a plateau that lasted 10 h and finally slightly decreased until the last measurement at 21 h. Regarding the mean mesh size, it continuously increased from 0 to 21 h. Based on these observations, we could assume that neovascularization begins by the formation of many vessels that branch to form a network for a certain time, 7 h in our case, then only the overall network evolves, with microvascular endothelial cells moving to create a more harmonized net pattern with regular meshes. 

For further use of this model and to test our sulfated polysaccharides, we defined 9.5 h as the incubation time after which the capillary network was well formed and stable at this time. Pictures taken at *t* = 9.5 h could then be compared to see the potent anti-angiogenic activities of the tested compounds.

### 2.4. Anti-Angiogenic Potential of Heparanase Inhibitors

After establishing a Matrigel^TM^ test implicating heparanase in the angiogenesis process, the anti-angiogenic potential of the LMW anti-heparanase polysaccharides we produced was assessed. Compounds were tested at a concentration of 200 µg/mL and their impact on pseudo-vessels formation and number of junctions in the angiogenesis network were measured. The previous kinetic study indicated that in the HskMEC Matrigel^®^ model, the angiogenesis tended to develop quickly and then mature, to form a regular net pattern. We then investigated on one hand, the effect of the LMW sulfated polysaccharides on the angiogenesis development during the first seven hours, when the cellular activity is the highest and, on the other hand, the number of pseudo vessels formed at *t* = 9.5 h, when angiogenesis reached a plateau. The rate of angiogenesis formation was represented as the slope of the linear regression made on the evolution, over time, of the number of pseudo vessels (from 0 h to 7 h) and junctions (1.5 h to 7 h) (slopes obtained are presented in Supplementary Materials).

**Figure 4:**
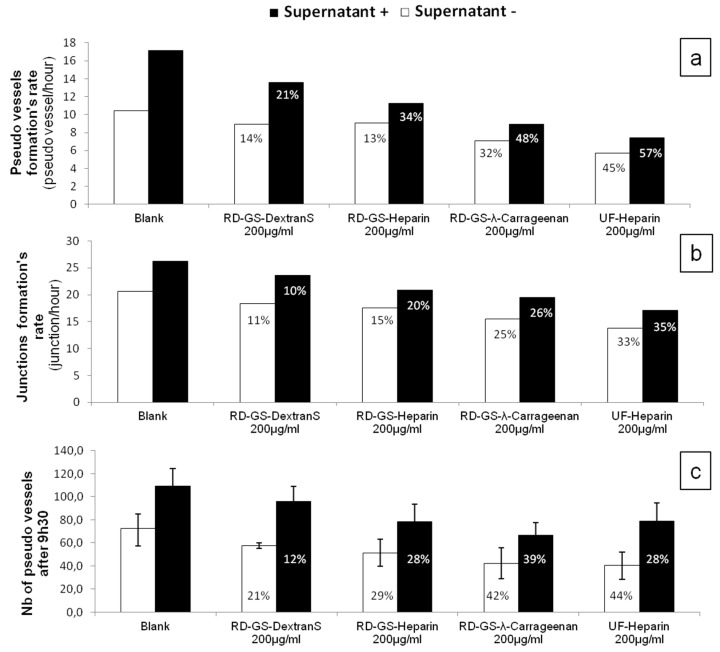
Effects of heparanase inhibitors on the kinetics of HSkMEC pseudovessels formation and junctions between them. Cells were incubated with heparanase inhibitors (200 µg/mL) on Matrigel either in the presence (black columns) or absence (white columns) of MCF-7 heparanase-rich supernatant. Angiogenesis kinetic was assessed by: the determination of pseudo-vessels formed between 0 and 7 h (**a**); and junctions formed between 1.5 h to 7 h (**b**) with photos taken every 30 min. Results are presented as the slope of a linear regression realized with number of pseudo vessels and junctions determined at each time with the Image J software (see Supplementary Materials). (**c**) The number of pseudo vessels (±SD) formed at *t* = 9.5 h. Inhibition of the angiogenesis development is specified for each compound tested and indicated as a percentage missing compared to the blank values. Complete kinetics from 0 to 19 h are presented in Supplementary Materials.

**Figure 5:**
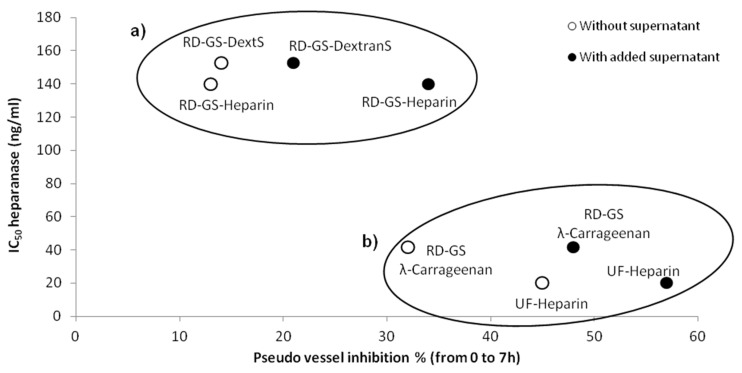
Comparison of the anti-angiogenic and anti-heparanase activities of studied sulfated polysaccharides. (**a**) The population comprising RD-GS-Heparin and RD-GS-DextranS has low anti-heparanase activity and anti-angiogenic activity. (**b**) The population comprising UF-Heparin and RD-GS-λ-Carrageenan has high anti-heparanase activity and high anti-angiogenic activity.

Overall, the four compounds slowed down the angiogenesis development, both in the FBS-free or in the MCF-7 induced tube formation (Figure 4). As shown in Figure 5, it appears that the more the compound inhibits heparanase, the more it slows the angiogenesis development. Thus, the RD-GS-λ-Carrageenan, proposed as a good alternative to heparin for heparanase inhibition, was able to slow the speed of formation of pseudo vessels by 32% in FBS-free medium and 48% in heparanase-rich medium. In comparison, UF-heparin slowed the speed of formation of pseudo vessels by 45% in classic medium and 57% in heparanase-rich medium (Figure 4a).

When looking at the precise time (9.5 h) where angiogenesis has reached a plateau, the potential of the RD-GS-λ-Carrageenan seems confirmed (Figure 4c). Indeed, compared to the blank control, the number of pseudo vessels at 9.5 h is reduced by 39% in the presence of RD-GS-λ-Carrageenan in medium supplemented by MCF-7 supernatant when UF-heparin displayed a lower reduction of 28% in the same conditions. In this analysis, all the LMW sulfated polysaccharides present lower inhibition when MCF-7 supernatant was added. The most stricking examples concern UF-heparin and RD-GS-DextranS. They display an inhibition of pseudo vessels formation of respectively 44% and 21% when FBS-free medium is used and 28% and 12% when the supernatant is added. This could be explained because in classic medium, even in hypoxic conditions, the HsKMEC microendothelial cells did not secrete much heparanase (see Figure 2). However, when heparanase-rich medium from MCF-7 was added in the medium, the resulting higher level of enzyme made the inhibition less efficient as the same inhibitor concentrations were tested in both cases. 

Moreover, it is also well described that breast cancer cells can produce a variety of growth factors such as VEGF or FGF-2 [49,51]. These additional growth factors can as well stimulate angiogenesis and reduce the efficacy of tested compound, although heparin is known for binding to VEGF [52], and λ-carrageenan have the potential for binding to growth factors such as FGF-2 [24]. 

A correlation seems to appear between anti-heparanase activity and angiogenesis inhibition of our compounds (Figure 5). We thus hypothesized that they mainly regulate angiogenesis through interaction with heparanase. The endothelial cells mobility and invasion capacity are key factors for the formation of pseudo vessels and heparanase helps this phenomenon by degrading the HS component of the ECM (here the Matrigel) [53]. A low mobility of the cells was observed, along with the reduced capacity to form pseudovessels, when analyzing microscope photos of wells containing heparanase inhibitors such as UF-Heparin or RD-GS-λ-Carrageenan, supporting the prevalent role of heparanase in angiogenesis. 

Coupled with its low anti-coagulant activity [38], the RD-GS-λ-Carrageenan appeared as a promising anti-angiogenic candidate, although its inhibition potential both in absence or presence of MCF-7 supernatant may suggest that it is not specific to heparanase. In fact, other sulfated polysaccharide-based inhibitors of heparanase actually in clinical trial such as SST0001 or PG-545 do not exclusively inhibit heparanase but also interact with VEGFs or FGFs [54]. Moreover, our results support the recent study by Niu et al. [24] showing that λ-Carrageenan disaccharide structure bearing an average of three sulfates (d-galactose 2-sulfate + d-galactose 2,6-sulfate) is similar to HS structural features and can bind FGF-2 and inhibit heparanase. Its anti-angiogenic potential through inhibition of heparanase seems even more interesting as a recent modeling study showed its capacity to bind to the active site of heparanase [55].

## 3. Material and Methods

### 3.1. Cell Culture

The Human Skin Microvascular Endothelial Cell line (HSkMEC) was established in the Centre for Molecular Biophysics of Orléans according to the method previously described [29] and patented (C. Kieda, Centre national de la Recherche Scientifique, patent 99-16169). Their phenotype is stable in terms of adhesion molecules and endothelial cells characteristics [29]. Human mammary carcinoma cell line (MCF-7), and metastatic mammary carcinoma cell line MDA-MB-231 were purchased from ATCC.

Cell lines were cultured at 37 °C in OptiMEM with Glutamax I (ThermoFischer Europe, Paisley, Scotland, UK) supplemented with 2% heat-inactivated Fetal Bovine Serum (FBS) (Sigma Aldrich, St. Louis, MO, USA), 40 µg/mL gentamycin (ThermoFisher Europe, Paisley, Scotland, UK) and 0.05 µg/mL fungizone (ThermoFisher Europe, Paisley, Scotland, UK).

For hypoxia treatment, cells were placed in a humidified atmosphere at 37 °C with a stabilized gas mixture input containing 94%N_2_/5%CO_2_/1%O_2_ (Air Liquide, Paris, France) in a Hypoxystation^®^ H35. For normoxia treatment, cells were incubated in a humidified atmosphere maintained at 37 °C in 5% CO_2_/95% atmospheric air.

To simulate nutrient poor conditions, cultures were FBS-deprived. The cells were incubated for the first 24 h with a complete OPTIMEM medium supplemented with 2% FBS for a good adhesion and multiplication. Then the medium was replaced by complete FBS-free OPTIMEM medium and cells were incubated for 48 h before collecting supernatants.

### 3.2. Western Blotting

Cells were grown to 80% confluence and lysed in lysis buffer (50 mM Tris-HCl, pH 7.5, 100 mM NaCl, 5 mM EDTA, 50 mM NaF, 1% Triton X-100, 1X concentrated complete protease inhibitor cocktail (Sigma Aldrich Europe, Roche science, Mannheim, Germany) (25X corresponding to one tablet dissolved in 2 mL buffer) for 10 min on ice with regular stirring. Cells were finally centrifuged at 10,000 g for 10 min at 4 °C and supernatants were stored at −80 °C.

Protein concentration was determined using the DC protein assay (BioRad, Hercules, CA, USA), an improved version of the well-documented Lowry assay.

Rabbit anti-human Heparanase was purchased from AbCam (Cambridge, UK), rabbit anti-human actin was purchased from Cell signaling technology (Danvers, MA, USA) and goat anti-rabbit IgG peroxidase was purchased from Life technology (Paisley, Scotland, UK).

Proteins from cell lysate samples (22 µg) were separated by SDS-PAGE on 15% gel, using precision plus protein dual color standard (Bio-Rad, Hercules, CA, USA) as molecular-weight size markers, and electroblotted on PVDF membranes. These membranes were first incubated with the primary antibody (rabbit anti human actin diluted 1:1000 or rabbit anti-human heparanase diluted 1:500) in 0.05% TBS-Tween with 10% dried milk for 1 h at room temperature. Then, the membranes were washed 5 times with 0.05% TBS-Tween before incubation with the secondary antibody (anti-rabbit IgG peroxidase diluted 1:33) in 0.05% TBS-Tween with 10% dried milk for 1 h at room temperature. Proteins were detected by chemoluminescence (West Dura supersignal detection system) using Pxi equipped with GeneSys software.

### 3.3. ELISA Assay

The heparanase release profiles in HSkMEC, MCF-7 and MDA-MB-231 cell line supernatants were analyzed using Human HPSE/Heparanase ELISA kit according to the manufacturer’s instructions (LSBio, Seattle, WA, USA).

### 3.4. In Vitro Angiogenesis Assay

Ninety-six-well plates were coated with growth factor-reduced Matrigel^TM^ (BD Bioscience, Allschwil, Switzerland). Cells were trypsinized, washed and seeded at 15,000 cells per well in FBS-free medium. Inhibitors were added at 200 µg/mL final concentration. The experiment was performed in two conditions, in the presence of 10 µL of MCF-7 heparanase-rich supernatant (10% of final volume) or without (replaced by FBS-free culture medium). Cells were cultured under hypoxic (1% O_2_) conditions. Cell rearrangement and pseudo-vessel formation were visualized using a Zeiss Axiovert 200M fluorescence inverted microscope (Zeiss, Le Pecq, France) equipped with an Axiocam high-resolution numeric camera connected to a computer driving the acquisition software Axiovision (Zeiss, Le Pecq, France). The direct real-time visualization of the pseudo-vessel formation was monitored for 24 h. Photographs were taken, processed using Axiovison Software and analyzed with ImageJ software with the Angiogenesis analyzer add-on [56]. Data obtained allowed angiogenesis quantification through determination of pseudo-vessel number, inter-vessel junction number and mesh network number.

### 3.5. Elaboration of Sulfated Polysaccharides

Heparins, Dextran sulfate (shortened DextranS in this paper) and λ-Carrageenan were purchased respectively from Alfa Aesar, Sigma Aldrich and FMC Biopolymer. The respective modified molecules were called RD-GS-Heparin, RD-GS-DextranS and RD-GS-λ-Carrageenan.

The RD-GS prefix denotes the radical-assisted depolymerization (RD) of the commercial polysaccharides associated with glycol splitting (GS).

#### 3.5.1. Physicochemical Depolymerization of Polysaccharides by Ultrasonic-Assisted Radical Depolymerization

Polysaccharides were dissolved in water to a final concentration of 25 mg/mL (up to 4 mL) for Heparin and dextranS and 5 mg/mL for λ-Carrageenan due to its low solubility. The reaction mixture was sonicated using a 3-mm diameter probe-type UP50H ultrasonicator (Hielscher, Teltow, Germany) (micro tip MS3), which provided an amplitude of 180 µm. The processor generated mechanical longitudinal vibrations with a frequency of 30 kHz that were produced by electrical excitation. With the reaction mixture at room temperature, the sonic pulses maintained a temperature of 60 °C in the reactor. Hydrogen peroxide 30% was added to obtain a final hydrogen peroxide/polysaccharide (*w*/*w*) ratio of 0.15. After 8 h of depolymerization, the reaction mixture was lyophilized prior to Glycol splitting.

#### 3.5.2. Glycol-Split Polysaccharide Preparation

Glycol-split polysaccharides were prepared by exhaustive periodate oxidation followed by borohydride reduction [18]. Reaction mixtures (3 mL) containing 40 mg of previously depolymerized polysaccharides and 0.1 M NaIO_4_ were incubated in the dark at 4 °C under stirring at 500 rpm. After a 12 h incubation, 120 µL of ethylene glycol were added to neutralize the excess of periodate. The incubation conditions were the same as above for 1 h. Then, 26 mg of solid NaBH_4_ were added to the reaction mixture at 4 °C and the reaction was stirred for 4 h to let the temperature return to ambient. The pH was then adjusted to 4 with 1 M HCl before neutralization with 1 M NaOH. The mixtures were finally desalted by dialysis using a 1kDa porous cellulose ester membrane (spectra/Por Dialysis membrane Molecular weight cut-off (MWCO): 1000 Da). Dialysis was performed 3 times for 12 h at 4 °C against 4 L of ultrapure water.

### 3.6. SEC-HPLC Analysis

Structural and quantitative analyzes of reaction products by size exclusion chromatography (SEC) were performed using an Agilent LC/MS-ES system (Santa Clara, CA, USA) (1100 LC/MSD Trap VL mass spectrometer) with one TSK-GEL G5000PW and one TSK-GEL G4000PW column (Tosoh bioscience, Griesheim, Germany) mounted in series. Columns were maintained at 30 °C and the products were eluted with 0.1 M sodium nitrate (NaNO_3_) at a flow rate of 0.5 mL/min. Products were detected and quantified by differential refractometry using HP Chemstation software off-line for the processing (Agilent, Santa Clara, CA, USA).

Heparin oligosaccharides of different molecular weights purchased from Iduron (Manchester, UK) were used as calibrants for the standard curve and calculation of the size of heparin derivative products. Dextrans of different molecular weights purchased from Sigma Aldrich were used as calibrants for the standard curve and calculation of the size of DextranS and Carrageenan derivatives.

Number-averaged molar mass (M*_n_*) was calculated as follows [57]:

M*_n_* = (∑N*_i_* × M*_i_*)/∑N*_i_* (1)

where N*_i_* is the number of moles of polymer species, and M*_i_* is the molecular weight of polymer.

### 3.7. Cell Viability Assay

Sulfated polysaccharides were tested on HSkMEC cultures to measure their cytotoxicity using Alamar Blue^®^ assay. This assay was performed over 4 days. On Day 1, cells were seeded into a 96-well plate at 500 cells/well in 50 µL of complete OPTIMEM medium supplemented with 2% FBS and incubated for 24 h at 37 °C. On Day 2, 40 µL of complete OPTIMEM medium containing different concentrations of sulfated polysaccharides were added into the wells. Concentrations were tested in 8 replicates. The plate was incubated for another 24 h at 37 °C. On Day 3, 10 µL of commercial solution of Alamar Blue^®^ (Life technology, Paisley, Scotlant, UK) were added into each well and the plate was incubated for another 24 h at 37 °C. On Day 4, each well supernatant was transferred into a black polystyrene plate (Corning^®^ #3915, Kennebunk, ME, USA) adapted for fluorescence and measurements were made using the following parameters: λ_ex_ = 544 nm and λ_em_ = 590 nm with a BMG Labtech Fluostar Omega spectrofluorometer (BMG Labtech, Ortenberg, Germany).

### 3.8. Sulfate Determination

The stability of the sulfate groups on the sugar backbone was monitored using (7-aminophenothiazin-3-ylidene)-dimethylazanium chloride (Azure A), which binds the sulfated groups in a polysaccharide chain [58]. Briefly, 200 µL of a 10 mg/L solution of aqueous Azure A were added to 20 µL of diluted sulfated polysaccharide samples. Absorbance was then measured at 535 nm. Dextran sulfate with characterized sulfur content was used to build the calibration curve. The sulfur content was then transposed into sulfate content.

### 3.9. Heparanase Inhibition Assay

Heparanase inhibition assays were performed using the heparanase assay toolbox (Cisbio Assay, Codolet, France) with heparanase purchased from R&D systems (human recombinant heparanase HPSE). The hydrolysis of Biotin-H-Eu(K) (heparan sulfate labeled with both biotin and Eu3+ cryptate) was performed in white 96-well half-area plates (Corning^®^ #3693) using a BMG Labtech Fluostar Omega spectrofluorometer with a Homogenous time resolved fluorescence (HTRF) module (BMG Labtech, Ortenberg, Germany). The reaction was performed in the presence or the absence of polysaccharide products at 37 °C in a final volume of 60 µL. First, 15 µL of inhibitor solutions in ultrapure water were added into the wells followed by 15 µL of heparanase solution (400 ng/mL heparanase in Tris-HCl pH 7.5, 0.15 M NaCl and 0.1% CHAPS). After 10 min of preincubation at 37 °C, an enzyme reaction was initiated by adding 30 µL of Bio-HS-Eu(K) (4.4 ng/µL in 0.2 M Sodium acetate buffer, pH 5.5) and the plate was incubated at 37 °C for 15 min. Then 20 µL of strepavidin-d2 (10 ng/µL in 0.1 M NaPO_4_ buffer, pH 7.4, 0.8M KF, 0.1% BSA, 1 mg/mL Heparin) were added and incubated for 2 min at 37 °C. The fluorescence was measured at λ_em1_ = 620 nm and λ_em2_ = 665 nm, after 60 µs of excitation at λ_ex_ = 337 nm. The delta F (%) was calculated using the following equation as indicated by the suppliers:

(2) $$\mathrm{Delta}\;F\;(\%)=\frac{\left(F665\;/\;F620\right)\;sample\;–\;\left(F665/F620\right)\;blank}{\left(F665/F620\right)\;blank}\;\times \;100\%$$

where *F*665 and *F*620 are fluorescence signals measured at 665 nm and 620 nm, respectively.

IC_50_ were determined based on the curve fitting tool from SigmaPlot software (Systat Software Inc., San Jose, CA, USA) using the equation: Sigmoidal; logistic; 3 parameters.

### 3.10. Statistical Analysis

Data are presented as the mean ± SE of at least one triplicate run. For cell viability and angiogenesis characterization parameters (pseudo-vessels, junctions, and meshes), statistical analyses were performed using SigmaPlot software (Systat Software Inc., San Jose, CA, USA). Replicates (*n* = 7 for cell viability; *n* = 3 for angiogenesis parameters) were averaged and the values were tested for normality (Shapiro-Wilk test). Replicates were obtained from individual culture wells (*n* = 7 or *n* =3) and statistical significance was determined, performing an ANOVA test with the SigmaPlot software.

## 4. Conclusions

We herein investigated the heparanase inhibition potential of sulfated polysaccharides submitted to depolymerization and glycol splitting. The 11 kDa RD-GS-λ-Carrageenan produced had an interesting heparanase IC_50_ of 7.32 ng/mL. Then heparanase expression profile of three different cell lines was investigated: one microvascular (HSk-MEC) and two mammary carcinoma cell lines (MDA-MB-231 and MCF-7). We showed that hypoxia associated with nutrient-poor conditions were the best culture conditions for MCF-7 cells to excrete high amounts of mature heparanase. We used the resultant heparanase-rich supernatant to study its effect on the angiogenesis network formed by HSk-MEC in Matrigel^TM^. Results showed that its addition doubled the number of pseudo-vessels and meshes formed while the junctions were multiplied by 1.5 confirming the pro-angiogenic role of the enzyme. Finally, we assessed the anti-angiogenic properties of produced sulfated polysaccharides in the same Matrigel^TM^ model. An overall anti-angiogenic effect was observed for the four compounds and we hypothesized that it was related to their capacity to inhibit heparanase, although it remains unclear whether the tested polysaccharides acted solely on heparanase or also by blocking growth factors or their receptors.

Among the four sulfated polysaccharides tested, the 11 kDa RD-GS-λ-Carrageenan emerged as a promising anti-angiogenic candidate, as it was able to inhibit pseudo-vessel formation by 42% in the absence of MCF-7 heparanase-rich supernatant and 39% in its presence. It also displayed a capacity to slow the angiogenesis process by reducing the formation of pseudo vessels by 32% for the seven first hours in an in vitro Matrigel^®^ test. This λ-carrageenan derivative produced by a simple physico-chemical process offers new options in terms of medical use for this family of sulfated polysaccharide extracted from red algae and mostly exploited today by the food industry.

In a further study, we plan to investigate more exhaustively the composition of the MCF-7 heparanase-rich supernatant used here, especially the growth factors content, and to determine the ability for the 11 kDa RD-GS-λ-Carrageenan to interact with them as well as with heparanase.

## Figures and Tables

**Table 1:** Physicochemical characterization and biological activities of UF-Heparin and RD-GS polysaccharides. (a) Sulfation degree = mol sulfates/mol disaccharide. (b) Heparanase inhibition expressed as IC_50_ values (ng/mL) of inhibitors against heparanase (100 ng/mL). (c) Survival of HSkMEC cells exposed to compounds concentration of 250 µg/mL in complete OPTIMEM medium supplemented with 2% FBS for 48 h. * *p* ≤ 0.05.

Polysaccharides	Mn (Da)	Sulfation Degree ^a^	Heparanase Inhibition ^b^ (IC_50_ Values in ng/mL)	HSkMEC Cell Viability ^c^
UF-Heparin	16,412	2	10.7	68.9 ± 13.9% *
RD-GS-Heparin	2716	1.4	147	85 ± 9% *
RD-GS-DextranS	4710	0.9	61.5	84.2 ± 5.8% *
RD-GS-λ-Carrageenan	11,396	1.1	7.32	89.2 ± 6.2% *

## Supplementary Materials

The following are available online at www.mdpi.com/1660-3397/15/5/134/s1, Figure S1: Complete Kinetic of the number of pseudo vessels formation from 0 h to 20 h, Figure S2: Complete Kinetic of the junctions formation from 0 h to 20 h, Figure S3: Linear regression of the number of junctions formed between 0 h and 7 h (supernatant +), Figure S4: Linear regression of the number of junctions formed between 0 h and 7 h (supernatant -), Figure S5: Linear regression of the number of pseudo vessels formed between 0 h and 7 h (supernatant +), Figure S6: Slopes determined with linear regression of the number of pseudo vessels formed between 0 h and 7 h (supernatant).
