# Supplementary material for: Assessment of Heparanase-Mediated Angiogenesis Using Microvascular Endothelial Cells: Identification of λ-Carrageenan Derivative as a Potent Anti Angiogenic Agent
Source: Mar Drugs. 2017 May 9;15(5):134. doi: 10.3390/md15050134 (PMC5450540; doi:10.3390/md15050134)
Supplement: Supplementary file 1 [file marinedrugs-15-00134-s001.pdf]

Assessment of Heparanase-Mediated Angiogenesis using Microvascular Endothelial Cells: Identification of λ-Carrageenan Derivative as a Potent Anti Angiogenic Agent

Nicolas Poupard [1], Pamela Badarou [2], Fabienne Fasani [2], Hugo Groult [1], Nicolas Bridiau [1], Frédéric Sannier [1], Stéphanie Bordenave-Juchereau [1], Claudine Kieda [2], Jean-Marie Piot [1], Catherine Grillon [2], Ingrid Fruitier-Arnaudin [1,*,†] and Thierry Maugard [1,†]

[1] Université de la Rochelle, UMR CNRS 7266, LIENSs, Equipe Approches Moléculaires, Environnement-Santé, Avenue Michel Crépeau, 17000 La Rochelle, France; nicolas.poupard@univ-lr.fr (N.P.); hugo.groult@univ-lr.fr (H.G.); nicolas.bridiau@univ-lr.fr (N.B.); frederic.sannier@univ-lr.fr (F.S.); stephanie.bordenave@univ-lr.fr (S.B.); jean-marie.piot@univ-lr.fr (J.P.); thierry.maugard@univ-lr.fr (T.M.);

[2] Centre de Biophysique Moléculaire, UPR CNRS 4301, 45071 Orléans, France; ayobadarou@hotmail.fr (P.B.); fabienne.fasani@cnrs-orleans.fr (F.F.); claudine.kieda@cnrs-orleans.fr (C.K.); catherine.grillon@cnrs-orleans.fr (C.G.)

* Correspondence: ingrid.fruitier@univ-lr.fr (I.F.); Tel.: +33-546-458-562; Fax: +33-546-458-277

† These authors contributed equally to this work.

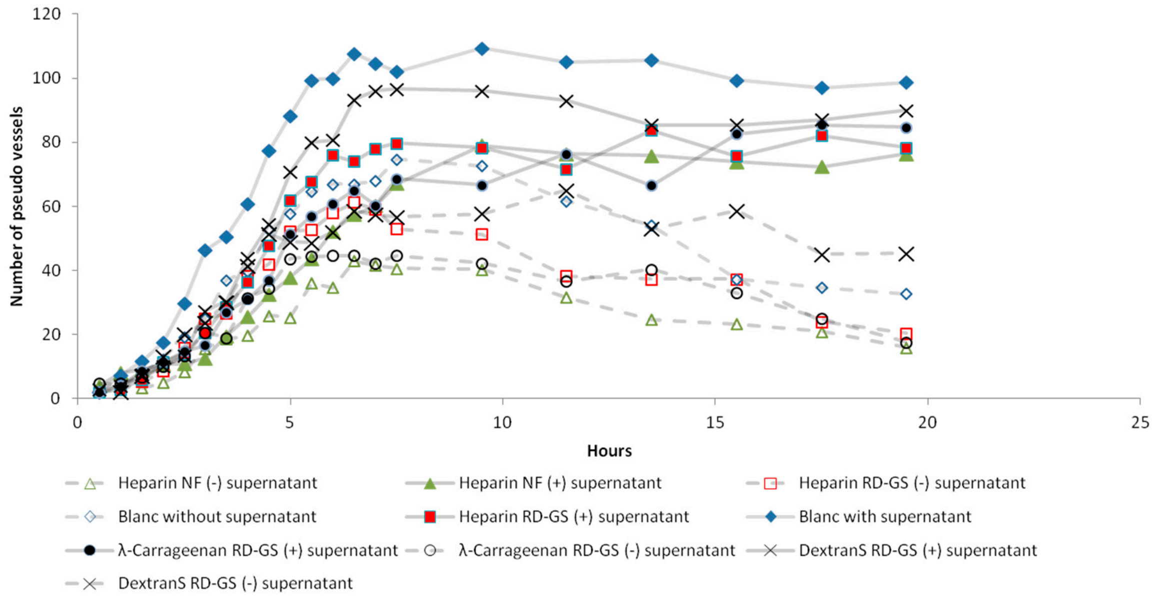

Figure S1: Complete Kinetic of the number of pseudo vessels formation from 0h to 20h. Each polysaccharide is described in the conditions with FBS-free medium ((-) supernatant) or with medium supplemented with MCF-7 supernatant ((+) supernatant). Each point represent the average value of a triplicate

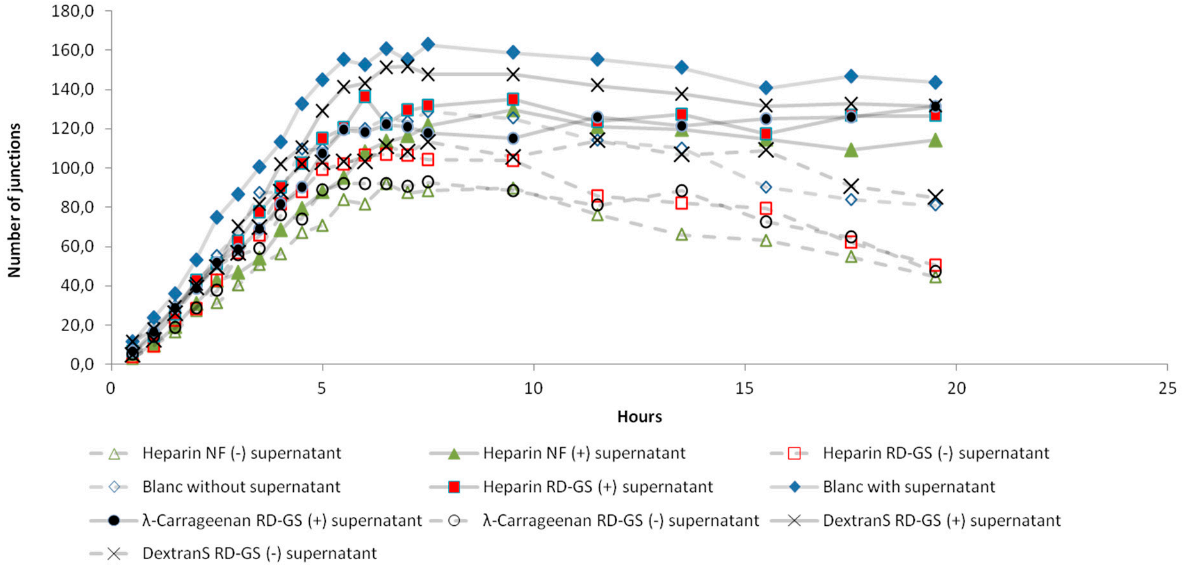

Figure S2: Complete Kinetic of the junctions formation from 0h to 20h. Each polysaccharide is described in the conditions with FBS-free medium ((-) supernatant) or with medium supplemented with MCF-7 supernatant ((+) supernatant). Each point represent the average value of a triplicate

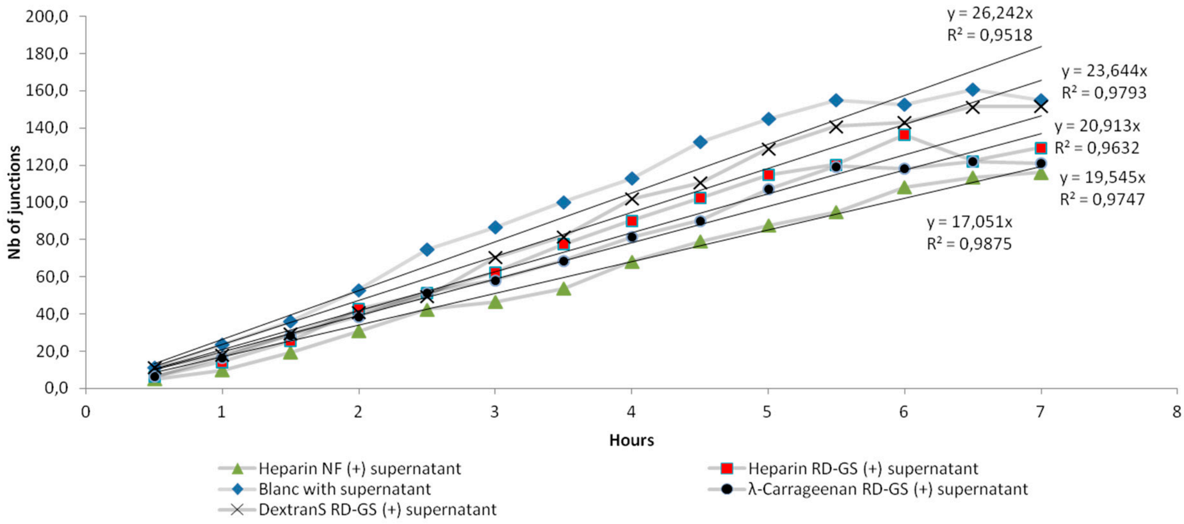

Figure S3: Slopes determined with linear regression of the number of junctions formed between 0h and 7h with each polysaccharide tested in medium supplemented with MCF7-supernatant. Each point represent the average value of a triplicate

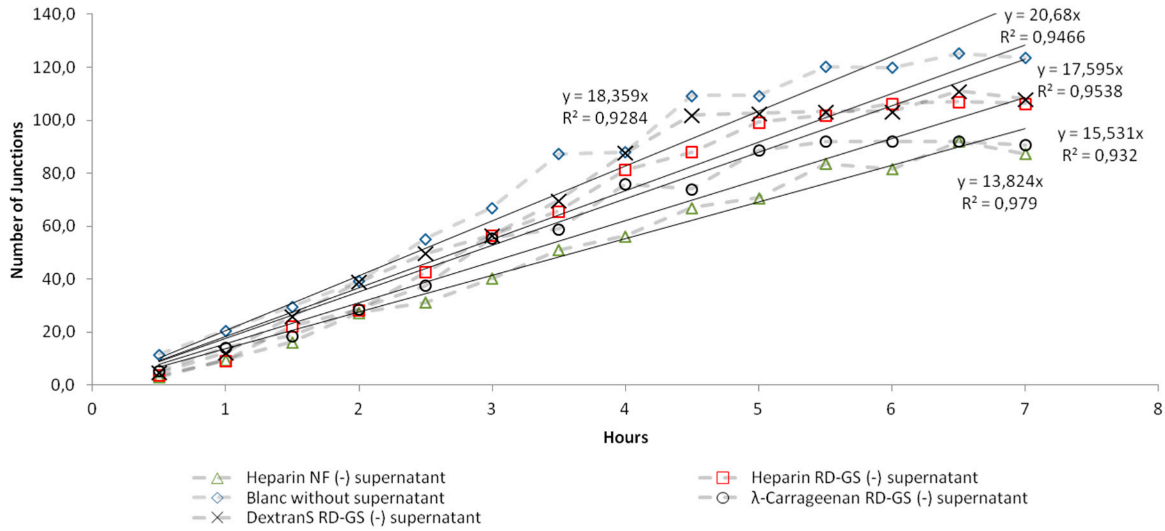

Figure S4: Slopes determined with linear regression of the number of junctions formed between 0h and 7h with each polysaccharide tested in FBS-free medium. Each point represent the average value of a triplicate

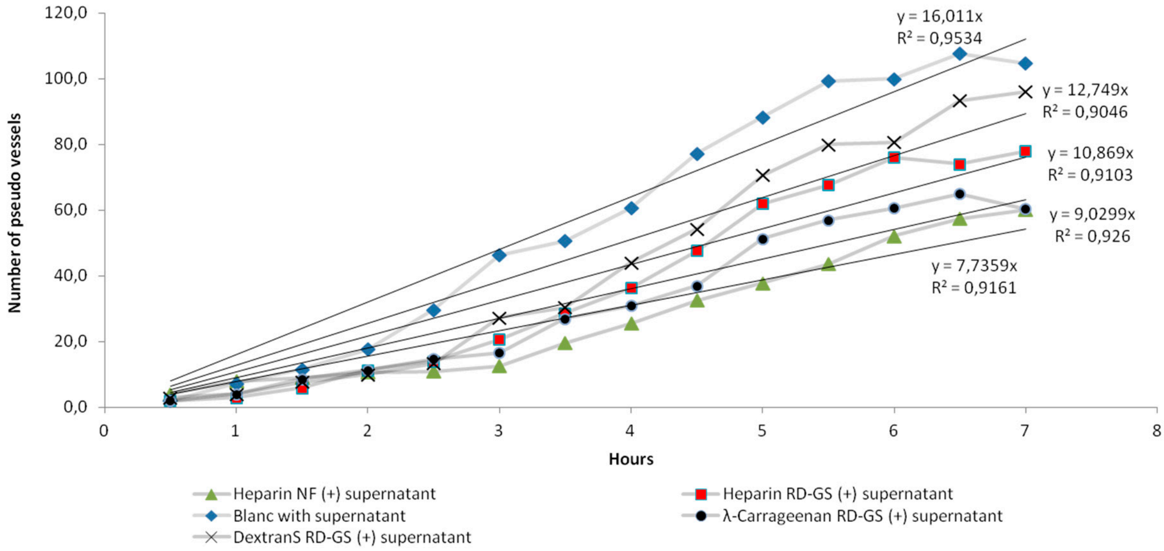

Figure S5: Slopes determined with linear regression of the number of pseudo vessels formed between 0h and 7h with each polysaccharide tested in medium supplemented with MCF7-supernatant. Each point represent the average value of a triplicate.

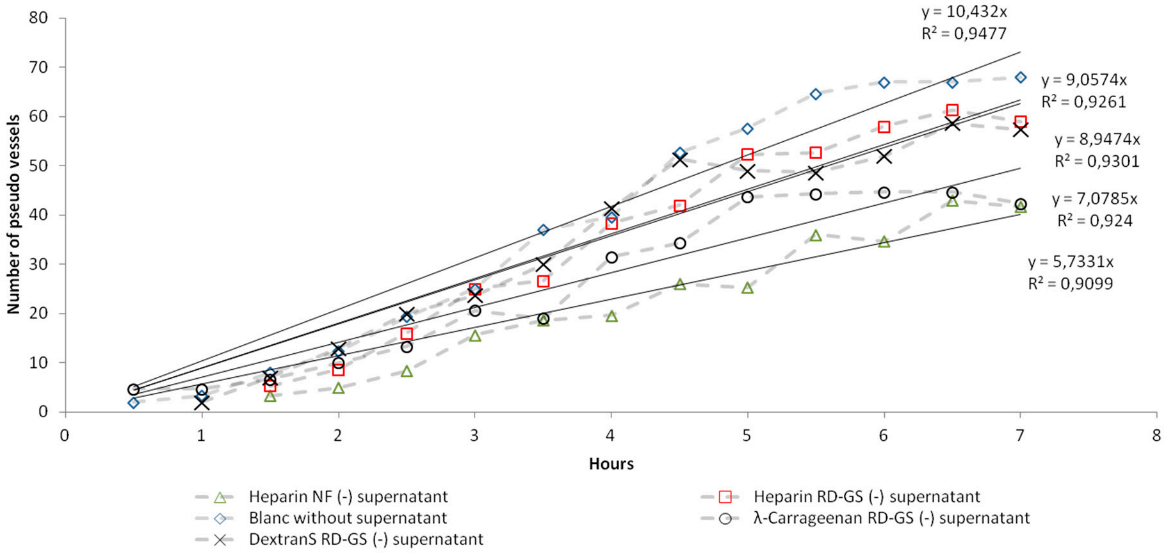

Figure S6: Slopes determined with linear regression of the number of pseudo vessels formed between 0h and 7h with each polysaccharide tested in in FBS-free medium. Each point represent the average value of a triplicate
